# How robust is the own-group face recognition bias? Evidence from first- and second-generation East Asian Canadians

**DOI:** 10.1371/journal.pone.0233758

**Published:** 2020-05-29

**Authors:** Andy H. Ng, Jennifer R. Steele, Joni Y. Sasaki, Meghan George

**Affiliations:** 1 Cardiff Business School, Cardiff University, Cardiff, Wales, United Kingdom; 2 Department of Psychology, York University, Toronto, Ontario, Canada; 3 Department of Psychology, University of Hawaiʻi at Mānoa, Honolulu, Hawaii, United States of America; Bournemouth University, UNITED KINGDOM

## Abstract

There is mounting evidence that North Americans are better able to remember faces of targets who belong to the same social group, and this is true even when the social groups are experimentally created. Yet, how Western cultural contexts afford the development of this own group face recognition bias remains unknown. This question is particularly important given that recent findings suggest that first-generation East Asian Canadians do not show this bias. In the current research, we examined the own-group bias among first- and second-generation East Asian Canadians, who vary systematically in their exposure to and engagement in a Western cultural context, and tested mediators that could explain any difference. In Study 1, second-generation East Asian Canadians showed better memory for same-group (vs. other-group) faces. In Studies 2 and 3, as well as a meta-analysis of all three studies, we found some additional evidence that second-generation East Asian Canadians show better memory for same-group (vs. other-group) faces, whereas first-generation East Asian Canadians do not, but only when each cultural group was examined separately in each study, as no interaction with generational status emerged. In Study 2, and in a higher powered pre-registered Study 3, we also examined whether second- (vs. first-) generational status had a positive indirect effect on same-group face recognition through the effects of acculturation and perceived relational mobility in the immediate social environment, however this mediation model was not supported by the data. Overall, the results provide some additional evidence that the effect of mere social categorization on face recognition may not be as consistently found among East Asian participants.

## Introduction

A substantial amount of research has confirmed that social categorization affects face recognition biases, with targets who belong to the same social group being more easily remembered than those who belong to a different social group (see [1] for a review). Initial research suggests, however, that this own-group bias in face recognition is not universal, with East Asians failing to show similar enhanced face recognition for same-group targets [2]. The goal of the current research was to further our understanding of face recognition biases by examining how the own-group bias might differ as a function of cultural context and testing two potential mediators of this difference: acculturation and perceived relational mobility.

### The own group face recognition bias

There is growing empirical evidence that North Americans are more motivated to remember faces of strangers who belong to the same social group, as opposed to a different social group. For instance, MacLin and Malpass [3] manipulated the hairstyle of ambiguous Hispanic–Black faces to make the faces appear to belong to different racial categories. They found that Hispanic Americans were better at recognizing these faces when they were perceived as racial ingroup members (i.e., with a Hispanic hairstyle) than when the identical faces were perceived as belonging to a racial outgroup (i.e., with a Black hairstyle). Moreover, Shriver, Young, Hugenberg, Bernstein, and Lanter [4] found that face recognition of racial ingroup targets decreased when the targets were perceived as belonging to a socio-economic outgroup. That is, when middle-class European American participants viewed pictures of White targets, their recognition performance was higher when the same targets were portrayed as coming from a middle-class (vs. working-class) background. Likewise, Bernstein, Young, and Hugenberg [5] demonstrated that merely categorizing novel faces into an ingroup or an outgroup using a minimal group paradigm influenced people’s face recognition performance. Specifically, European American participants were randomly assigned to either a “red” or a “green” group based on the results of a bogus personality test. Novel White targets were then presented against either a red or a green background, which purportedly represented the targets’ personality group. Targets who participants believed belonged to the same (vs. different) personality group were better recognized (cf. [6]).

Although mounting evidence suggests that this own-group bias in face recognition is common among European Americans, there is some evidence to suggest that it is not universal. Following the procedure of Bernstein and colleagues [5], Ng and colleagues [2] found in two studies that 1^st^ generation East Asian Canadians (born in East Asia but currently living in Canada) did not demonstrate the same own-group bias; their face recognition accuracy for ingroup and outgroup strangers did not differ. This was in contrast to European Canadians, who demonstrated an own-group bias. Furthermore, these findings were not moderated by the race of the targets, which included both East Asian and White faces that were equally represented in the experimentally created ingroups and outgroups. The finding that the own-group bias does not occur among participants of East Asian cultural backgrounds, in contrast to those of Canadian [2] and American [5] cultural backgrounds, is important as it may help increase our understanding of the underlying reason for this face recognition bias.

### Why are people motivated to remember same-group strangers?

Humans have a strong tendency to form and maintain interpersonal relationships [7]. Whether others are classified as potential partners, however, may depend on self–other similarities, as people are generally more attracted to others who are similar to the self [8]. People may be more likely to retain the identity, and specifically remember faces, of novel social targets who belong to the same social group because shared social group membership can signal similarity between the perceiver and the target, which can in turn signal the value of the target as someone with whom one may want to interact and potentially develop a relationship in the future. This possibility has indeed received empirical support. Wilson, See, Bernstein, Hugenberg, and Chartier [9] found that European Americans believe that they will have more interactions with other people who belong to the same (vs. different) group in the future. However, when this belief is manipulated such that people expect equal interactions with same-group and other-group targets, the own-group bias disappears. As such, the own-group bias seems to be a face memory bias that may serve a social connection function, facilitating the recognition of novel social targets who are perceived as worthy of approach, interaction, and potential relationship formation in the future. If the own-group bias in face recognition is a psychological tendency that emerges to facilitate future interaction and relationship formation with others who share similar characteristics, why do people of East Asian cultural backgrounds not show this same bias?

A review of the cultural psychology literature suggests that, across a number of domains, East Asians are less likely to demonstrate own-group biases than are North Americans. For example, researchers found that North American students hold more positive attitudes toward other students who attend the same (vs. different) university, whereas Japanese students do not show this own-group positivity bias [10]. In addition, using a minimal group paradigm, American participants allocated a larger amount of monetary bonus to ingroup versus outgroup members, whereas Japanese participants did not [11]. Cultural differences in these own-group biases have been explained by cultural differences in self-enhancement. That is, motivations for positive self-views are more pervasive for people in individualistic (e.g., North American) cultures than for people in collectivistic (e.g., East Asian) cultures (see [12] for a meta-analytic review), resulting in more positive evaluative and behavioral responses toward social groups to which an individual belongs in individualistic (vs. collectivistic) cultures. Although self-enhancement motivations can explain attitudinal and behavioral discrepancies between the ingroup and an outgroup, it seems a less likely explanation for cultural differences in face recognition biases.

Instead, there is an emerging literature suggesting that culture-contingent behaviors may also reflect strategies adapted in response to perceived relational mobility in the socio-ecological context. Relational mobility is the ease with which individuals can form new interpersonal relationships and terminate old ones (see [13] for a review). At the national level, people who live in Western European and North American countries (e.g., United Kingdom, United States) perceive that their social environments are relationally more mobile, affording more opportunities to meet new people and form relationships, compared with people who live in East Asian countries (e.g., China, Japan) [14, 15, 16]. For example, when asked about their perception of others in their social context using the Relational Mobility Scale [17], Japanese (vs. American) participants were less likely to endorse items such as “It is easy for them to meet new people” and “They can choose who they interact with” [15]. Perceived relational mobility also varies within the same country, including Japan [18] and Canada [19], and across situations or life stages, such as between first- and second-year university students [20].

Perceived relational mobility may be a useful lens through which to interpret within- and between-culture differences, particularly for psychological tendencies central to interpersonal behaviors. When people perceive that their social contexts are highly relationally mobile, psychological tendencies that increase the chances of acquiring new desirable relationship partners tend to emerge, such as trusting other people [21], seeking uniqueness as relationship “selling points” [22], and having relatively high self-esteem [23]. By contrast, when people perceive low relational mobility, they tend to demonstrate psychological tendencies that help them avoid relationship deterioration, such as less willingness to disclose personal information to close friends to avoid negative evaluations [24], experiencing shame in the company of close friends [19], and being sensitive to social rejection in general [25].

We propose that when people perceive that their social environments are relationally mobile, they should be motivated to individuate others, particularly those who share a broad social group membership (e.g., attending the same university), because of the potential for ingroup members to serve as future relationship partners. Comparatively, when people perceive that their social environments are low in relational mobility, they should have a tendency to focus on maintaining pre-existing social networks. Consequently, they should have less motivation to individuate strangers, who may be perceived as socially irrelevant even when they share a broad social group membership.

### The present research

The goal of this research was to examine cultural differences in the own-group bias in face recognition and to test whether perception of relational mobility afforded by Western cultures contributed to this potential difference. Given that people with the same heritage cultural background can have very different experiences in the mainstream cultural context, we focused on participants who shared the same heritage cultural background (i.e., East Asian) but varied in their exposure to and engagement in a Western (Canadian) cultural context. East Asian Canadians who were born and raised in Canada (i.e., 2^nd^ generation) should be more acculturated to the mainstream European Canadian culture [26, 27] and accordingly, might be more likely to share the mainstream perception of high relational mobility, compared with East Asian Canadians who were born in East Asia (i.e., 1^st^ generation) and thus have lived in Canada for a shorter period of time (cf. [19]). We expected that these differences in relational mobility would have consequence for the own-group bias.

Across all three studies, we examined whether 2^nd^ generation East Asian Canadians, who should have greater exposure to the mainstream European Canadian culture because they were born and raised in Canada, would demonstrate an own-group bias, as has been found consistently with other North American samples [2, 4, 5], but has not been found among 1^st^ generation East Asian Canadians [2]. In Studies 2 and 3, we examined the face recognition biases of 1^st^ generation East Asian Canadians as well and anticipated that they would *not* show an own-group bias. In Studies 2 and 3 we also tested possible factors that might contribute to the different pattern of results for 1^st^ and 2^nd^ generation East Asian Canadians–specifically, mainstream Canadian acculturation and perceived relational mobility in the immediate social environment.

## Study 1

The goal of Study 1 was to examine the own-group bias among 2^nd^ generation East Asian Canadians using an experimentally manipulated social group [2, 5]. Although race of target was not central to our research question, we employed the same method as previous research [2]–using both White and East Asian faces–to examine whether the own-group bias in face recognition toward the same experimentally manipulated social group would generalize to targets of different races. We hypothesized that 2^nd^ generation East Asian Canadians would exhibit the own-group bias (i.e., ingroup faces would be better recognized than outgroup faces), similar to others in their mainstream cultural context (i.e., European Canadians, European Americans) [2, 5] rather than their heritage cultural background (i.e., 1^st^ generation East Asian Canadians) [2]. Moreover, we expected that the own-group bias exhibited by 2^nd^ generation East Asian Canadians would not be moderated by target race, consistent with what has been found among European Canadians [2, cf. 4].

### Method

#### Ethics statement

This study was approved by the Institutional Review Board at York University (ethics approval number: e2014–226). Each participant read an informed consent form on a computer screen and indicated their consent by pressing a designated key on a computer keyboard prior to participation.

#### A priori power analysis

Using the effect size of Bernstein and colleagues (2007; Study 2), power analysis indicated that 45 participants were required to achieve a power of .80. We conducted this study in one semester and worked to recruit as many 2^nd^ generation East Asian Canadian participants as possible. As the number of participants was very close to the required number, we decided to conclude the study at the end of the semester.

#### Design and participants

Forty-one 2^nd^ generation East Asian Canadian university students (25 female; *M*_age_ = 19.3 years), defined as those who self-identified as East Asian and were born in Canada, completed a purported study of personality and face perception for course credit. This study had a 2 (Target Race: White vs. East Asian) × 2 (Target Group: Ingroup vs. Outgroup) within-subjects design.

### Materials

#### Personality test

We followed the procedure of Bernstein and colleagues [2, 5] to experimentally manipulate social groups, asking participants to complete 40 questions from the Big Five Personality Test [28], ostensibly to measure their personality type.

#### Face stimuli

As in Ng et al. [2], we used 120 grey-scaled face photographs of White (*n* = 60) and East Asian (*n* = 60) male targets as stimuli [29, 30, 31], with each face showing a neutral expression. Each face photograph was 6 × 5.25 inches and appeared on either an orange or green background; half of the faces of each race appeared on each color background. All face photographs were counterbalanced across background colors.

### Procedure

Consenting participants completed the personality test on a computer. They were then led to believe that their responses were analyzed by the computer, providing ostensible results indicating that the participant belonged to either an “orange” or a “green” personality group. Participants’ “personality” group was actually randomly assigned. They were then asked to wear either an orange or a green wristband corresponding to their personality group and were reminded that the wristband identified them as a member of their group.

Next, participants viewed 60 face photographs on the computer screen, including 30 White (half with orange and half with green backgrounds, counterbalanced) and 30 East Asian (half with orange and half with green backgrounds, counterbalanced) faces, presented individually and in random order. As such, each face photograph was equally likely to be associated with either an orange or green background. Each face photograph remained on the screen for 3 seconds with an inter-stimulus interval of 0.5 seconds. Participants were told to pay attention to the photos, as their memory of these faces would be tested.

After a 5-minute filler task, participants viewed 120 faces presented sequentially in the center of the computer screen. These 120 faces included the 60 old faces on their original background color and 60 new faces (30 White and 30 East Asian) presented equally on either an orange or green background, counterbalanced. As such, each of the new face photograph was equally likely to be associated with either an orange or green background. For each face, participants were asked to indicate, by pressing one of two keys, whether they had seen it previously. Each face remained on the screen until the participant responded. Finally, participants answered demographic questions (e.g., gender, age, race) before being debriefed.

### Results and discussion

Face recognition accuracy scores were computed using the signal detection parameter sensitivity (*d’*) [32], where *d’* = *z*(hit)–*z*(false alarms) for each of the four groups of face targets (two target races crossed with two personality groups). The two personality groups were then recoded into “ingroup” and “outgroup” based on each participant’s own personality group assignment. For each participant, there were four *d'* scores representing face recognition accuracy for White ingroup faces, White outgroup faces, East Asian ingroup faces, and East Asian outgroup faces. We identified one outlier (> 3*SD*s above the mean) for White-Outgroup recognition, which was removed before conducting analyses.

**Fig 1:**
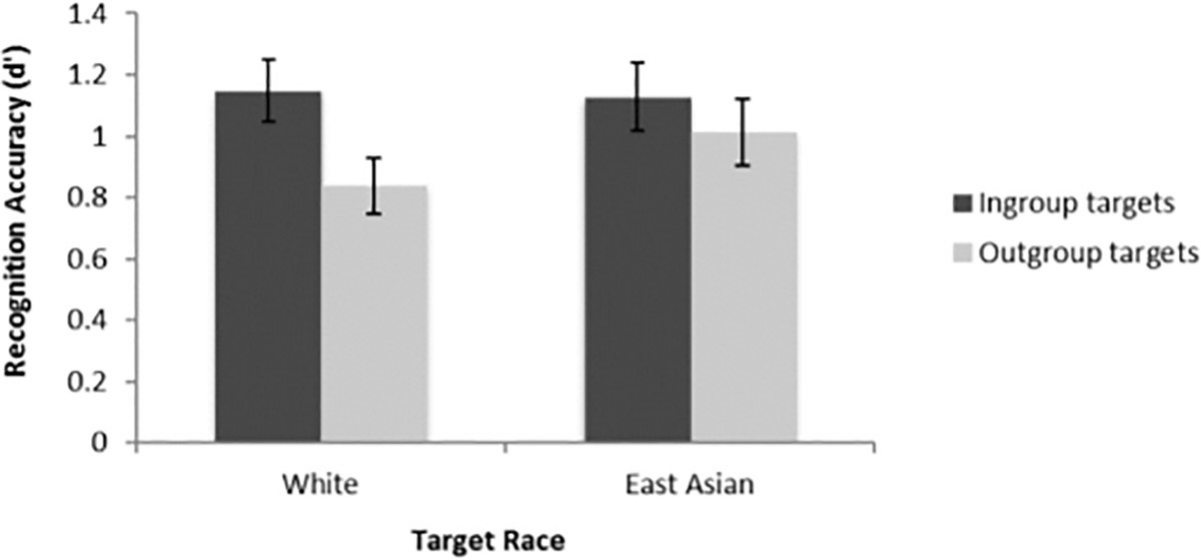
Face recognition accuracy in Study 1 as a function of target race and target group (personality group) among 2^nd^ generation East Asian Canadian participants. Error bars indicate standard errors.

As hypothesized, a 2 (Target Race: White vs. East Asian) × 2 (Target Group: Ingroup vs. Outgroup) repeated-measures ANOVA (see Table 1 for *M*s and *SD*s in each condition) revealed a significant main effect of Target Group. Second generation East Asian Canadian participants showed better memory for ingroup faces (*M* = 1.14, *SD* = 0.55), based on the personality group membership, over outgroup faces (*M* = 0.93, *SD* = 0.52), *F*(1, 39) = 5.33, *p* = .03, $\eta_{p}^{2}$ = .12, 95% CI of the difference: [.03, .40] (see Fig 1). The main effect of Target Race was not significant, *F*(1, 39) = 0.76, *p* = .39, $\eta_{p}^{2}$ = .02. The interaction effect between Target Race and Target Group was also not significant, *F*(1, 39) = 1.76, *p* = .19, $\eta_{p}^{2}$ = .04.

**Table 1:** Mean face recognition accuracy (*d'*) scores in Study 1 (personality group) among 2^nd^ generation East Asian Canadian participants.

East Asian ingroup targets	1.13 (0.69)
East Asian outgroup targets	1.01 (0.68)
White ingroup targets	1.15 (0.64)
White outgroup targets	0.84 (0.57)

Standard deviations are shown in parentheses.

In Study 1 we replicated the own-group bias in face recognition among 2^nd^ generation East Asian Canadians, demonstrating that targets who shared the same (vs. different) experimentally manipulated personality group were recognized more accurately, regardless of race. As predicted, East Asian Canadians who were born and raised in a North American context exhibit the own-group bias. This effect has been found previously among participants who share their Western cultural context, specifically among European Canadian and European American participants [2, 5] but not among participants who share their Eastern heritage cultural background (i.e., 1^st^ generation East Asian Canadians [2]). Taken together with our previous research, in which we found that the own-group bias did not generalize to 1^st^ generation East Asian Canadian perceivers [2], the present results suggest that the mainstream North American cultural context may enhance people’s motivation to memorize faces of strangers who share the same social group membership. The specific aspect of North American cultural context that might explain this difference was examined in Study 2.

While the present findings demonstrate the own-group bias in 2^nd^ generation East Asian Canadian participants, this group did *not* show better memory for same-race (vs. cross-race) faces. While initially this finding might seem at odds with the robust cross-race effects typically found in the broader literature on face recognition [33], this is consistent with some previous research on cross-race face recognition among East Asian participants [34] who do not always show a cross-race effect. We revisit this point in the General Discussion.

## Study 2

The first goal of Study 2 was to conceptually replicate the findings of Study 1 using a larger sample of participants and a pre-existing broad social group that has also been used in previous research–university affiliation [2, 5]. Specifically, we examined whether 2^nd^ generation East Asian participants would again show the own-group bias when presented with faces who purportedly belonged to either their ingroup or their outgroup. To accomplish this, participants from York University, a large university located in central Canada, were randomly assigned to a color condition and were led to believe that faces presented on the same color background were students from York University. Participants were told that faces on a different color background were students from the University of Calgary, a university of a comparable size, located in western Canada. We hypothesized that, as in Study 1, 2^nd^ generation East Asian Canadians would show an own-group bias, with enhanced memory for faces that purportedly shared their university affiliation emerging relative to faces that do not. Moreover, in Study 2 we also recruited 1^st^ generation East Asian Canadian participants and anticipated that we would replicate the previous finding [2] that 1^st^ generation East Asian Canadians do not show an own-group bias.

The second goal of Study 2 was to directly examine whether mainstream acculturation as well as perceived relational mobility might contribute to face memory for strangers who share the same broad social group membership of university affiliation. We tested a mediation model in which the effect of generational status (1^st^ or 2^nd^ generation) on face memory for same-group targets (targets who purportedly had the same university affiliation as the participants) was mediated sequentially by mainstream acculturation and perceived relational mobility. We hypothesized that East Asian Canadians who were born and raised in Canada (vs. East Asia) would be more acculturated to mainstream (European Canadian) culture.

Moreover, we expected that the more East Asian Canadians had acculturated to the mainstream (European Canadian) culture, the more likely they would be embedded in mainstream European Canadian environments and share the same perception of relational mobility with European Canadians. As noted earlier, there are both cultural and individual differences in the extent to which people believe that they have opportunities to meet new people and establish new relationships, with people from Western cultures typically perceiving greater relational mobility in their social environments than those from East Asian cultures [14, 15, 16]. As such, we anticipated that acculturation would predict perceived relational mobility, with those with higher levels of acculturation perceiving greater relational mobility in their social environments. Finally, given the motivational nature of the own-group bias [33], we expected that perceived relational mobility would positively predict memory for faces that purportedly shared the same university affiliation.

### Method

#### Ethics statement

This study was approved by the Institutional Review Board at York University (ethics approval number: e2014–226). Each participant read an informed consent form on a computer screen and indicated their consent by pressing a designated key on a computer keyboard prior to participation.

#### A priori power analysis

Using the effect size of Ng and colleagues [2], power analysis indicated that a total of 142 participants were required to achieve a power of .80 to detect the hypothesized moderating effect of generational status on the own-group bias. We conducted this study in two semesters and aimed to recruit as many 1^st^ and 2^nd^ generation East Asian Canadian participants as possible to account for potential dropouts after predetermined exclusion criteria (see below).

#### Design and participants

A total of 161 students from York University participated in the present study. We excluded participants a priori who had one or more friends (*n* = 14) or close family members (*n* = 16) in Calgary, or who did not correctly recall the color that was associated with York University in the experiment (*n* = 13; seven participants fell in more than one of these categories; see [2] for similar exclusions). The responses from the remaining 125 participants were retained. Seventy-seven of these participants were 2^nd^ generation East Asian Canadians, defined as those who self-identified as East Asian and were born in Canada (38 female; *M*_age_ = 18.7 years). An additional 48 participants were 1^st^ generation East Asian Canadians (29 female; *M*_age_ = 20.9 years), defined as those who self-identified as East Asian and were born in an East Asian country. For these 1^st^ generation East Asian Canadians, they had lived in Canada for 8.3 (*SD* = 6.15) years on average.

### Materials

#### Face stimuli

The face stimuli used in this study were identical to those used in Study 1.

#### Acculturation

Acculturation was measured using 16 items from the 20-item Vancouver Index of Acculturation (VIA) [27]. The VIA has two subscales–mainstream acculturation (α = .86) and heritage acculturation (α = .86)–and uses a 9-point response scale (1 = *strongly disagree*, 9 = *strongly agree*). Although our hypothesis concerned mainstream acculturation only, we administered both subscales for the sake of completeness. Sample items for the mainstream acculturation subscale include: “I often participate in mainstream Canadian cultural traditions” and “It is important for me to maintain or develop mainstream Canadian cultural practices”. Two parallel items from each subscale were not administered because we either felt that it might be less relevant for our student sample with limited work experience (“I am comfortable working with Canadians (people who are East-Asian)”) or that their responses might reflect an American influence and not acculturation (“I enjoy Canadian (East Asian) entertainment.”).

#### Perceived relational mobility

Perceived relational mobility was measured using the 12-item Relational Mobility Scale (RMS) [17] in which participants indicated the extent to which they agreed to some statements describing the *people around them in the immediate social environment where they lived* (α = .57). The RMS uses a 6-point response scale (1 = *strongly disagree*, 6 = *strongly agree*). Sample items include: “They have many chances to get to know other people” and “It is common for these people to have a conversation with someone they have never met before”.

### Procedure

The procedure was similar to that of Study 1 with the following exceptions. First, instead of completing a personality test, participants were randomly assigned to wear either an orange or a green wristband. Participants were told that the color represented York University and the wristband was used to identify them as a member of York University. Participants were instructed that they would view face photos of York University and University of Calgary students, and the background color of the photos (counterbalanced as in Study 1) would identify the university affiliation of the targets. Second, after completing the face recognition test, participants completed the VIA and the RMS. At the end of the study, participants were asked to recall the color that was associated with York University in the experiment, and indicate the number of friends they had at the University of Calgary and whether they had any close family member in Calgary for data exclusion purposes noted earlier. Finally, participants provided the same demographic information outlined in Study 1.

### Results and discussion

#### Moderating effect of generational status on own-group face recognition bias

As in Study 1, face recognition accuracy scores were computed using the signal detection parameter sensitivity (*d’*) [32] for each of the four groups of face targets, specifically White ingroup (same-university) faces, White outgroup (other-university) faces, East Asian ingroup faces, and East Asian outgroup faces. We identified one outlier (> 3*SD*s above the mean) for White-Ingroup recognition for the 2^nd^ generation group, which was removed prior to conducting analyses.

We conducted a 2 (Generational Status: 1^st^ vs. 2^nd^) × 2 (Target Race: White vs. East Asian) × 2 (Target Group: Ingroup vs. Outgroup) mixed ANOVA, with the first factor between-subjects and the last two factors within-subjects (see Table 2 for *M*s and *SD*s in each condition). The main effect of Target Group was not significant, *F*(1, 122) = 1.22, *p* = .27, $\eta_{p}^{2}$ = .01. However, in this study, the main effect of Target Race was significant, *F*(1, 122) = 20.85, *p* < .001, $\eta_{p}^{2}$ = .15, with East Asian faces (*M* = 1.01, *SD* = 0.48) being recognized more accurately than White faces (*M* = 0.76, *SD* = 0.56), 95% CI of the difference: [.14, .36]. The interaction effect between Generational Status and Target Group was not significant, *F*(1, 122) = 2.45, *p* = .12, $\eta_{p}^{2}$ = .02, and there were no other significant effects, *F*s < 2.06, *p*s > .15, $\eta_{p}^{2}s$ *<* .02.

**Table 2:** Mean face recognition accuracy (*d'*) scores in Study 2 (University affiliation) among 2^nd^ generation and 1^st^ generation East Asian Canadian participants.

	2^nd^ Generation Participants	1^st^ GenerationParticipants
East Asian ingroup targets	1.07 (0.62)	1.09 (0.75)
East Asian outgroup targets	0.89 (0.59)	0.99 (0.56)
White ingroup targets	0.83 (0.74)	0.69 (0.76)
White outgroup targets	0.69 (0.70)	0.84 (0.72)

Standard deviations are shown in parentheses.

**Fig 2:**
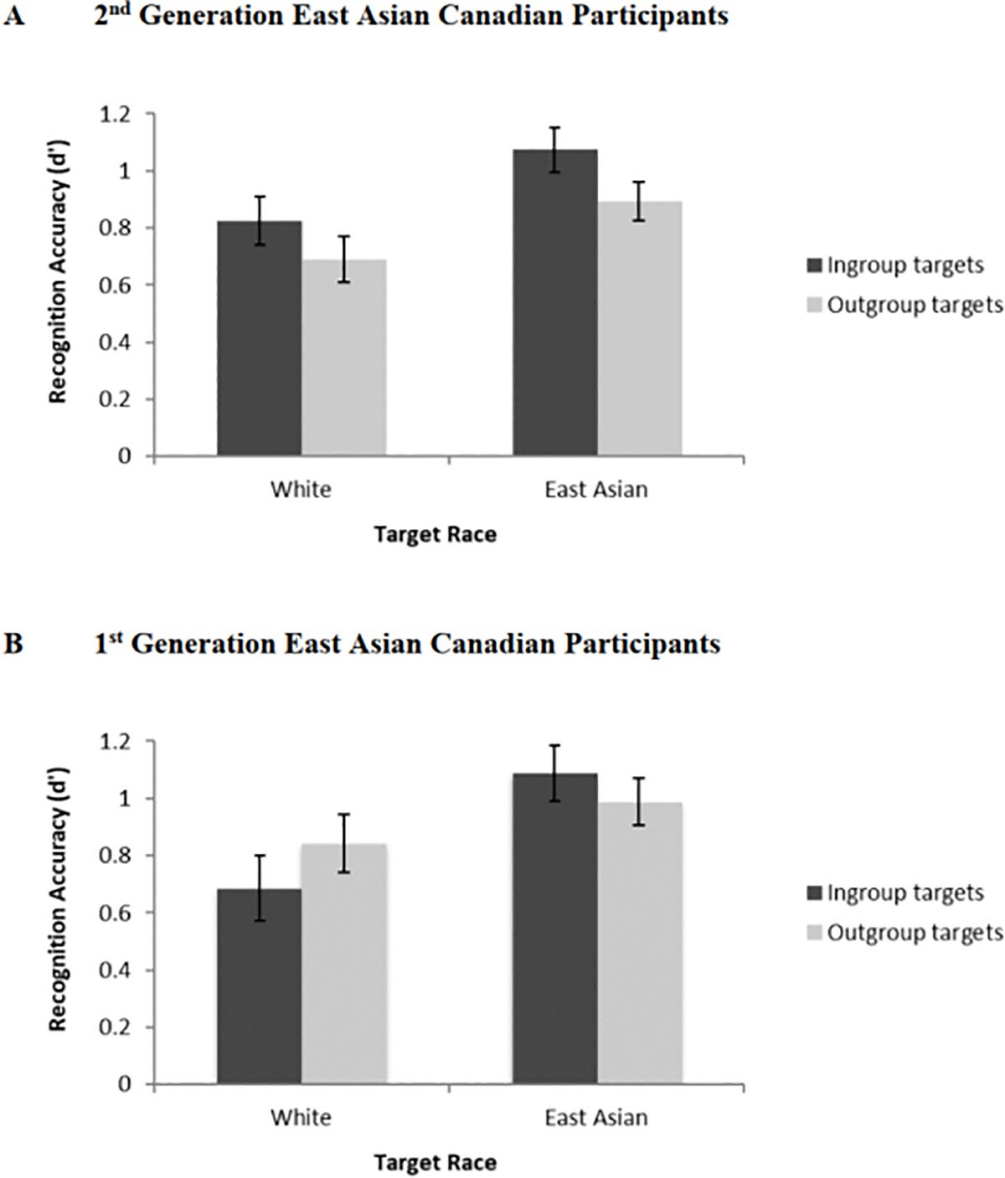
Face recognition accuracy in study 2 as a function of target race and target group (university affiliation) among (A) 2^nd^ generation East Asian Canadian and (B) 1^st^ generation East Asian Canadian participants. Error bars indicate standard errors.

Despite this lack of significant interaction, we conducted a 2 (Target Race: White vs. East Asian) × 2 (Target Group: Ingroup vs. Outgroup) within-subjects ANOVA within each generational group to test our specific hypotheses that 2^nd^ generation East Asian Canadians would show an own group bias whereas 1^st^ generation East Asian Canadians would not. For 2^nd^ generation East Asian Canadians, as expected, the main effect of Target Group was significant, *F*(1, 75) = 5.05, *p* = .03, $\eta_{p}^{2}$ = .06; they showed better memory for ingroup faces (*M* = 0.95, *SD* = 0.56) based on university affiliation over outgroup faces (*M* = 0.79, *SD* = 0.48), 95% CI of the difference: [.02, .30] (see Fig 2A), supporting our hypothesis and conceptually replicating Study 1. The main effect of Target Race was also significant, *F*(1, 75) = 10.64, *p* < .01, $\eta_{p}^{2}$ = .12, with East Asian faces (*M* = 0.98, *SD* = 0.45) being recognized more accurately than White faces (*M* = 0.76, *SD* = 0.57), 95% CI of the difference: [.14, .36]. Finally, the interaction effect between Target Race and Target Group was not significant, *F*(1, 75) = 0.12, *p* = .73, $\eta_{p}^{2}$ < .01.

For 1^st^ generation East Asian Canadians, as anticipated, and replicating previous research [2], the main effect of Target Group was not significant, *F*(1, 47) = 0.08, *p* = .78, $\eta_{p}^{2}$ < .01; their recognition for ingroup (*M* = 0.89, *SD* = 0.62) and outgroup faces (*M* = 0.92, *SD* = 0.53) did not differ significantly (see Fig 2B). The main effect of Target Race was significant, *F*(1, 47) = 10.73, *p* < .01, $\eta_{p}^{2}$ = .19, with East Asian faces (*M* = 1.04, *SD* = 0.54) being recognized more accurately than White faces (*M* = 0.77, *SD* = 0.54), 95% CI of the difference: [.11, .44]. Finally, the interaction effect between Target Race and Target Group was not significant, *F*(1, 47) = 2.41, *p* = .13, $\eta_{p}^{2}$ = .05.

#### Mediating roles of mainstream acculturation and perceived relational mobility

**Fig 3:**
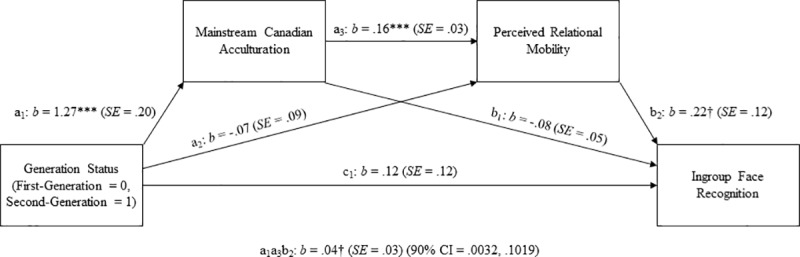
The serial mediation model of the relationship between generational status and ingroup face recognition in Study 2. †*p* < .10; *p < .05; **p < .01; ***p < .001.

We identified two outliers (> 3*SD*s below the mean) for mainstream acculturation for 2^nd^ generation participants, one outlier (> 3*SD*s below the mean) for perceived relational mobility for 2^nd^ generation participants, and two outliers (> 3*SD*s above the mean) for ingroup recognition for 2^nd^ generation participants. The four cases that contained these outliers (one case contained two of these outliers) were removed prior to conducting analyses. We tested our hypothesized mediation model (see Fig 3) that generational status would predict face recognition accuracy of targets who shared the same university affiliation through the effects of mainstream Canadian acculturation and perceived relational mobility in the immediate social environment, using a bootstrapping technique with 5,000 resamples [35] (see Table 3 for correlations among variables). Results indicated that, as expected, (a) generational status predicted acculturation to mainstream Canadian culture, *b* = 1.27 (*SE* = .20, 95% CI = .86, 1.67), β = .49 (*SE* = .08, 95% CI = .34, .65), *t*(119) = 6.19, *p* < .001; (b) mainstream Canadian acculturation predicted perceived relational mobility, *b* = .16 (*SE* = .03, 95% CI = .09, .22), β = .44 (*SE* = .10, 95% CI = .25, .63), *t*(118) = 4.51, *p* < .001; and (c) perceived relational mobility predicted ingroup face recognition, *b* = .22 (SE = .12, 95% CI = -.02, .46), β = .18 (*SE* = .10, 95% CI = -.02, .37), *t*(117) = 1.79, *p* = .08, although not at conventional levels of significance. The indirect effect of generational status on ingroup face recognition, mediated through the effect of Canadian acculturation and perceived relational mobility in serial was in the anticipated direction but not at conventional levels of significance, *b* = .04, *p* < .10, (*SE* = .03, 90% biased-corrected CI = .0032, .1019), β = .04 (*SE* = .03, 90% biased-corrected CI = .0028, .0930).

**Table 3:** Correlations among variables in Study 2.

	MCA	PRM	Ingroup Face Recognition
Generational Status	.49***	.14	.04
Mainstream Canadian Acculturation (MCA)		.40***	-.06
Perceived Relational Mobility (PRM)			.12

***p < .001 (two-tailed). For Generational Status, 1^st^ generation = 0, 2^nd^ generation = 1.

These findings suggest that, relative to 1^st^ generation East Asian Canadians, 2^nd^ generation East Asian Canadians are more inclined to associate with mainstream Canadians and engage in mainstream Canadian cultural practices, which in turn contributes to a perception of higher relational mobility of their immediate social environments. The data also hint at the possibility that these may, in turn, predict enhanced face memory of strangers who belong to the same social group; however given that these finding were in the anticipated direction but not statistically significant, any conclusions regarding this mediating process would be tentative at best. Given our sample size, it seemed possible that this model was not significant due to our study being underpowered. Therefore, in Study 3 we examined this mediation model again with a larger sample size.

## Study 3

The goal of Study 3 was to conceptually replicate the findings of Study 2 using personality type as the experimentally manipulated social group, in a higher powered study that was pre-registered prior to data collection (https://osf.io/um7qb). We hypothesized that, as in Study 2, 2^nd^ generation East Asian Canadians would show an own-group bias, with enhanced memory for faces that purportedly shared their personality type emerging relative to faces that do not, whereas 1^st^ generation East Asian Canadian participants would not show an own-group bias. Moreover, we tested the same mediation model as in Study 2 and hypothesized that the effect of generational status (1^st^ or 2^nd^ generation) on face memory for same-group targets (targets who purportedly had the same personality type as the participants) would be mediated sequentially by mainstream acculturation and perceived relational mobility.

### Method

#### Ethics statement

This study was approved by the Institutional Review Board at York University (ethics approval number: e2019-109). Each participant read an informed consent form on a computer screen and indicated their consent by pressing a designated key on a computer keyboard prior to participation.

#### A priori power analysis

Using the effect size of Study 2, an a priori power analysis indicated that 154 participants were required to achieve a power of .80 to detect the hypothesized interaction between participant generational status and target group on face recognition accuracy. As noted in our pre-registration, we aimed to recruit more than this required number of participants and decided a priori to recruit at least 240 participants (*n* = 120 1^st^ generation and *n* = 120 2^nd^ generation East Asian Canadian participants), so as to detect a potentially smaller effect size. Sensitivity power analysis indicates that, with *N* = 240, this study had a power of .80 to detect an effect size of $\eta_{p}^{2}$ = .01.

#### Design and participants

A total of 272 students from York University participated in the present study. We excluded participants a priori who reported that their country of origin was not Canada or an East Asian country (*n* = 6), or who did not correctly answer the manipulation check question “What is your personality type as revealed by the test that you did earlier?" (*n* = 24). The remaining 242 participants included 122 2^nd^ generation East Asian Canadians, defined as those who self-identified as East Asian and were born in Canada (75 female; *M*_age_ = 20.1 years), and 120 1^st^ generation East Asian Canadians (72 female; *M*_age_ = 23.2 years), defined as those who self-identified as East Asian and were born in an East Asian country. First generation East Asian Canadians had lived in Canada for 7.3 (*SD* = 6.69) years on average.

### Materials

#### Face stimuli

The face stimuli used in this study were identical to those used in Studies 1 and 2.

#### Acculturation

Acculturation was again measured using the Vancouver Index of Acculturation (VIA) [27] (α = .81 for mainstream acculturation; α = .87 for heritage acculturation), however unlike in Study 2, for completeness, we administered and included all items. Results remain comparable if only the items administered in Study 2 are retained.

#### Perceived relational mobility

As in Study 2, perceived relational mobility was measured using the 12-item Relational Mobility Scale (RMS) [17] (α = .63).

### Procedure

The face recognition test was identical to that of Study 1. After completing the face recognition test, participants completed the VIA and the RMS, and demographics questionnaire. At the end of the study, participants were asked to recall their personality type for data exclusion purpose noted earlier.

### Results and discussion

#### Moderating effect of generational status on own-group face recognition bias

As in Studies 1 and 2, face recognition accuracy scores were computed using the signal detection parameter sensitivity (*d’*) [32] for each of the four groups of face targets, specifically White ingroup (same-personality) faces, White outgroup (other-personality) faces, East Asian ingroup faces, and East Asian outgroup faces. We identified five outliers (> 3*SD*s above the mean)–one for White-Ingroup recognition for 1^st^ generation participants, one for White-Outgroup recognition for 1^st^ generation participants, one for White-Ingroup recognition for 2^nd^ generation participants, one for White-Outgroup recognition for 2^nd^ generation participants, and one for Asian-Outgroup recognition for 2^nd^ generation participants. These five outliers were removed prior to conducting analyses.

We conducted a 2 (Generational Status: 1^st^ vs. 2^nd^) × 2 (Target Race: White vs. East Asian) × 2 (Target Group: Ingroup vs. Outgroup) mixed ANOVA, with the first factor between-subjects and the last two factors within-subjects (see Table 4 for *M*s and *SD*s in each condition). Consistent with Study 2, the main effect of Target Group was not significant, *F*(1, 235) = 2.27, *p* = .13, $\eta_{p}^{2}$ = .01, and the main effect of Target Race was significant, *F*(1, 235) = 43.01, *p* < .001, $\eta_{p}^{2}$ = .16, with East Asian faces (*M* = 0.97, *SD* = 0.51) being recognized more accurately than White faces (*M* = 0.72, *SD* = 0.48), 95% CI of the difference: [.17, .32]. Unexpectedly, the hypothesized interaction effect between Generational Status and Target Group was not significant, *F*(1, 235) = 0.11, *p* = .74, $\eta_{p}^{2}$ < .01. However, a Target Race by Target Group interaction emerged, *F*(1, 235) = 4.70, *p* = .03, $\eta_{p}^{2}$ = .02. There were no other significant effects, *F*s < 0.47, *p*s > .49, $\eta_{p}^{2}s$ *<* .01.

**Table 4:** Mean face recognition accuracy (*d'*) scores in Study 3 (personality type) among 2^nd^ generation and 1^st^ generation East Asian Canadian participants.

	2^nd^ Generation	1^st^ Generation
	Participants	Participants
East Asian ingroup targets	0.97 (0.66)	0.94 (0.65)
East Asian outgroup targets	1.00 (0.58)	0.95 (0.65)
White ingroup targets	0.81 (0.71)	0.77 (0.59)
White outgroup targets	0.64 (0.61)	0.67 (0.59)

Standard deviations are shown in parentheses

**Fig 4:**
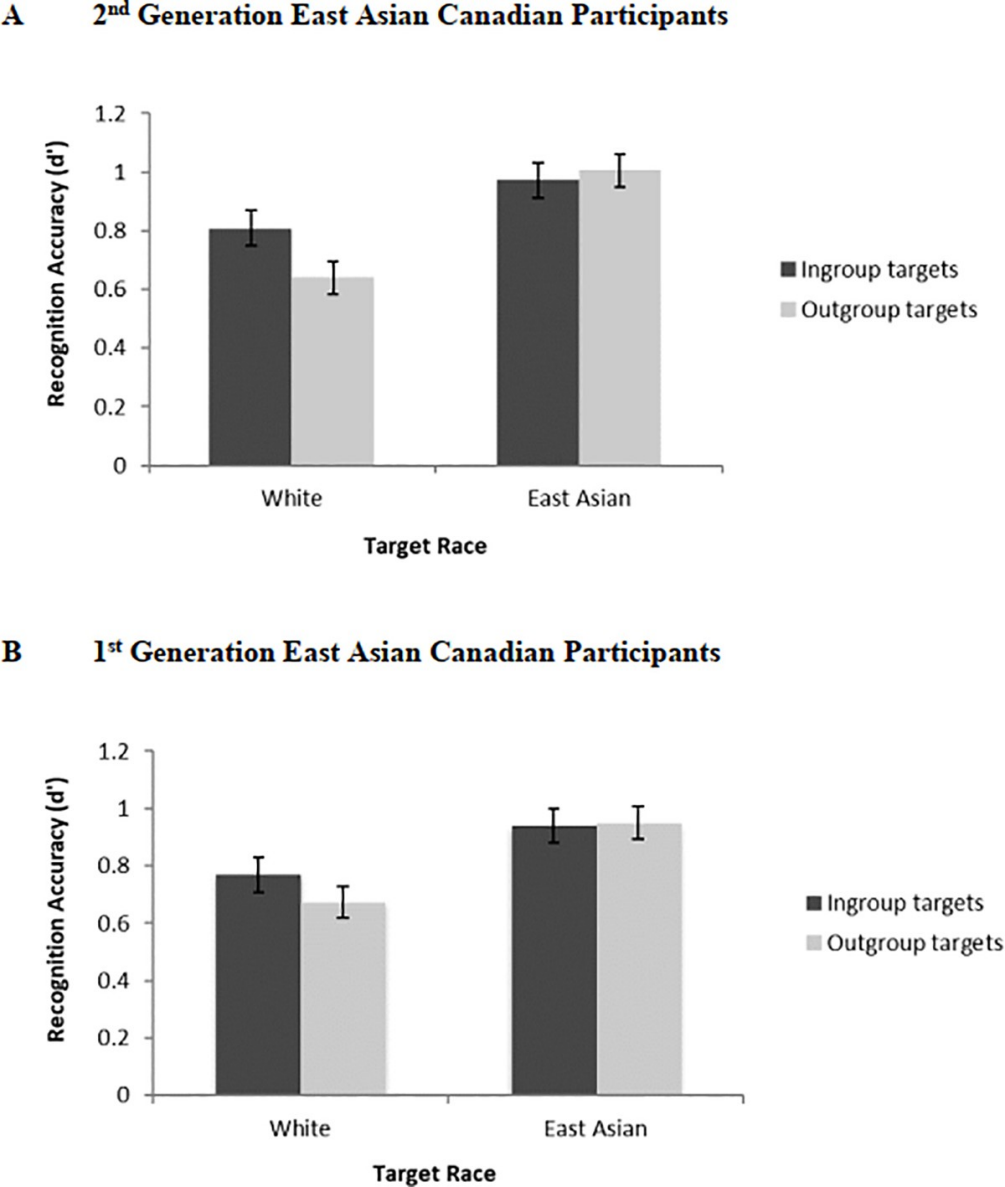
Face recognition accuracy in study 3 as a function of target race and target group (personality group) among (A) 2^nd^ generation East Asian Canadian and (B) 1^st^ generation East Asian Canadian participants. Error bars indicate standard errors.

As in Study 2, we conducted additional analyses, specifically a 2 (Target Race: White vs. East Asian) × 2 (Target Group: Ingroup vs. Outgroup) within-subjects ANOVA within each generational group, to test our specific hypotheses that 2^nd^ generation East Asian Canadians would show an own-group bias whereas 1^st^ generation East Asian Canadians would not, and to further explore the significant Target Race by Target Group interaction. For 2^nd^ generation East Asian Canadians, unlike Study 2, the main effect of Target Group was not significant, *F*(1, 118) = 1.69, *p* = .20, $\eta_{p}^{2}$ = .01; their recognition for ingroup (*M* = 0.89, *SD* = 0.51) and outgroup faces (*M* = 0.82, *SD* = 0.46) did not differ significantly. Instead, the interaction between Target Group and Target Race approached significance, *F*(1, 118) = 3.49, *p* = .06, $\eta_{p}^{2}$ = .03 and follow-up analyses suggest that 2^nd^ generation East Asian Canadians showed an own-group bias for White faces (ingroup recognition: *M* = 0.81, *SD* = 0.71; outgroup recognition: *M* = 0.64, *SD* = 0.61), *F*(1, 118) = 4.36, *p* = .04, $\eta_{p}^{2}$ = .04, 95% CI of the difference: [.01, .33], but not for East Asian faces (ingroup recognition: *M* = 0.97, *SD* = 0.66; outgroup recognition: *M* = 1.00, *SD* = 0.58), *F*(1, 118) = 0.23, *p* = .63, $\eta_{p}^{2}$ < .01 (see Fig 4A). The main effect of Target Race was significant, *F*(1, 118) = 23.81, *p* < .001, $\eta_{p}^{2}$ = .17, with East Asian faces (*M* = 0.99, *SD* = 0.49) being recognized more accurately than White faces (*M* = 0.72, *SD* = 0.49), 95% CI of the difference: [.16, .37].

For 1^st^ generation East Asian Canadians, as in Study 2, the main effect of Target Group was not significant, *F*(1, 117) = 0.69, *p* = .41, $\eta_{p}^{2}$ = .01; their recognition for ingroup (*M* = 0.86, *SD* = 0.49) and outgroup faces (*M* = 0.81, *SD* = 0.51) did not differ significantly (see Fig 4B). The main effect of Target Race was significant, *F*(1, 117) = 19.25, *p* < .001, $\eta_{p}^{2}$ = .14, with East Asian faces (*M* = 0.95, *SD* = 0.53) being recognized more accurately than White faces (*M* = 0.72, *SD* = 0.46), 95% CI of the difference: [.12, .33]. Finally, the interaction between Target Group and Target Race was not significant, *F*(1, 117) = 1.32, *p* = .25, $\eta_{p}^{2}$ = .01.

#### Mediating roles of mainstream acculturation and perceived relational mobility

**Fig 5:**
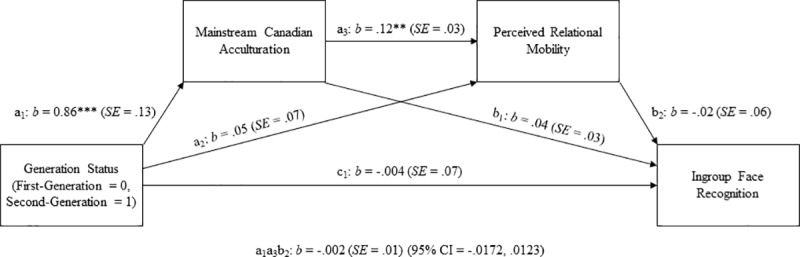
The Serial mediation model of the relationship between generational status and ingroup face recognition in Study 3. **p < .01; ***p < .001.

Although the hypothesized interaction effect between Generational Status and Target Group was not statistically significant, we examined our pre-registered hypothesized mediation model (same as Study 2, see Fig 5). We identified two outliers (> 3*SD*s above the mean) for ingroup recognition for 2^nd^ generation participants and one outlier (< 3*SD*s below the mean) for perceived relational mobility for 2^nd^ generation participants. The three cases that contained these outliers were removed prior to conducting analyses. We tested the mediation model using a bootstrapping technique with 5,000 resamples [35] (see Table 5 for correlations among variables).

**Table 5:** Correlations among variables in Study 3.

	MCA	PRM	Ingroup Face Recognition
Generational Status	.38***	.14*	.03
Mainstream Canadian Acculturation (MCA)		.26***	.08
Perceived Relational Mobility (PRM)			.00

***p < .001 (two-tailed)

*p < .05 (two-tailed). For Generational Status, 1^st^ generation = 0, 2^nd^ generation = 1.

Results indicated that generational status predicted acculturation to mainstream Canadian culture, *b* = 0.86 (*SE* = .13, 95% CI = .59, 1.12), β = .38 (*SE* = .06, 95% CI = .27, .50), *t*(237) = 6.41, *p* < .001, and mainstream Canadian acculturation predicted perceived relational mobility, *b* = .12 (*SE* = .03, 95% CI = .05, .18), β = .24 (*SE* = .07, 95% CI = .11, .38), *t*(236) = 3.55, *p* = .001. Contrary to our expectation, perceived relational mobility did not predict ingroup face recognition, *b* = -.02 (SE = .06, 95% CI = -.14, .11), β = -.02 (*SE* = .07, 95% CI = -.15, .11), *t*(235) = -0.28, *p* = .78. The hypothesized indirect effect of generational status on ingroup face recognition, mediated through the effect of Canadian acculturation and perceived relational mobility in serial was not significant, *p* > .05, *b* = -.002 (*SE* = .01, 95% biased-corrected CI = -.0172, .0123), β = -.002 (*SE* = .01, 95% biased-corrected CI = -.0169, .0122).

*Meta-analysis of generational differences in the own-group bias across studies*. As the format of all three of our studies had a similar structure, but some inconsistencies in the results, we conducted an internal meta-analysis using the procedure outlined in Goh, Hall, and Rosenthal [36] to further examine our main hypotheses regarding own-group bias for each of the generational groups. As the three studies were methodologically similar, a fixed effects approach was used to make inferences about them [37].

We first examined the own-group bias among 2^nd^ generation East Asian Canadian participants across studies. Focusing on this group of participants only and collapsing across the two target races, we conducted a dependent t-test to obtain the p-value and the effect size *r* of the own-group bias for each of the three studies with positive numbers indicating the own-group bias (ingroup faces were better recognized than outgroup faces) (Study 1: *t*(39) = 2.31, *p* = .03, effect size *r* = 0.35; Study 2: *t*(75) = 2.25, *p* = .03, effect size *r* = 0.25; Study 3: *t*(118) = 1.31, *p* = .19, effect size *r* = 0.12). All *r*s were Fisher’s z transformed for analyses and transformed back to *r*s for ease of interpretation. Across the three studies, the weighted mean effect size *r* of the own-group bias among 2^nd^ generation East Asian Canadian participants was *M*_*r*_ = .21. A summary p-value for the three studies was then calculated using the Stouffer formula [38]: *z* = 3.27, *p* < .01, two-tailed. This suggests that taken together across all three studies, 2^nd^ generation East Asian Canadians demonstrated the own-group bias.

Next, we examined the own-group bias among 1^st^ generation East Asian Canadian participants across studies. Focusing on this group of participants only and collapsing across the two target races, we conducted a dependent t-test to obtain the p-value and the effect size *r* of the own-group bias for each of the two studies with positive numbers indicating the own-group bias (ingroup faces were better recognized than outgroup faces) (Study 2: *t*(47) = -0.28, *p* = .78, effect size *r* = -0.04; Study 3: *t*(117) = 0.83, *p* = .41, effect size *r* = 0.08). All *r*s were then Fisher’s z transformed for analyses and transformed back to *r*s for ease of interpretation. Across the two studies, the weighted mean effect size *r* of the own-group bias among 1^st^ generation East Asian Canadian participants was *M*_*r*_ = .05. A summary p-value for the two studies was then calculated using the Stouffer formula [38]: *z* = 0.39, *p* = .70, two-tailed. This suggests that taken together the two studies, 1^st^ generation East Asian Canadians did not demonstrate the own-group bias.

## General discussion

In this research we investigated when the own-group face recognition bias does and does not persist, and we attempted to answer the question of why. Consistent with previous findings [2], we found in Studies 2 and 3 that 1^st^ generation East Asian Canadians did not show the own-group bias. A meta-analysis of the results of these two studies further supports this null finding. By contrast, in Study 1, we obtained evidence to suggest that the own-group bias in face recognition generalizes to a North American born, but non-European (i.e., East Asian) sample, specifically 2^nd^ generation East Asians. However, in Studies 2 and 3, this own-group bias in face recognition only emerged when the data of 2^nd^ generation East Asian participants were examined separately from those of 1^st^ generation East Asian participants, and in Study 3 only for White (but not Asian) targets. Given these results, we conducted a meta-analysis of the results of all three studies to further examine whether 2^nd^ generation East Asian Canadians showed the own-group bias in face recognition. The meta-analytic result suggests that, taken together, 2^nd^ generation East Asian Canadians demonstrate the own-group bias. These findings extend previous research by demonstrating that, similar to European Americans [5] and European Canadians [2], 2^nd^ generation East Asian Canadians also demonstrate the own-group bias in face recognition, presumably because of their exposure to and engagement in a Western cultural context. However, additional research suggests that 2^nd^ generation East Asian Canadians are also influenced by their Eastern cultural heritage, and there are a variety of ways in which these bicultural individuals may negotiate their two cultures [39]. Thus, whether and the degree to which these bicultural individuals show the own-group bias may be highly variable, and this could help to explain the variability in our results. Future research should explore other variables which might influence the propensity of 2^nd^ generation East Asian Canadians to exhibit the own-group bias.

In Studies 2 and 3, we also examined a possible mechanism underlying the effect of generational status on the own-group bias. Specifically, we tested the mediating roles of mainstream Canadian acculturation and perceived relational mobility. We anticipated that, relative to East Asian Canadian participants who were born in East Asia, those who were born in Canada would be more acculturated to mainstream European Canadian customs and practices and accordingly, they would be more likely to perceive greater relational mobility in their social environments, resulting in the development of a face recognition bias toward strangers who share a social group membership. In both studies, we found a relationship between generational status and acculturation, as well as acculturation and perceived relational mobility. However, perceived relational mobility did not predict ingroup face recognition, and the serial mediation model did not receive empirical support, suggesting that this is not the mediating mechanism for this difference. As such, future research will be needed to examine other cultural variables that might predict ingroup face recognition.

Although not the main focus of the present research, our stimuli, which included White and East Asian faces, allowed us to examine whether participants would show a memory bias favoring their racial ingroup, and to determine whether the own-group bias was moderated by the race of the target faces. We found that same-race (East Asian) targets were recognized more accurately than cross-race (White) targets in Studies 2 and 3, but not in Study 1, which included only 2^nd^ generation East Asian Canadian participants. For East Asian perceivers, although there is evidence that same-race faces are better recognized than cross-race faces [40, 41], it has also been documented that East Asian perceivers fail to show this cross-race face recognition bias [34]. In the present research, we suspect that our results for the cross-race face recognition bias, or lack thereof, reflect sample-specific experience in processing East Asian versus White faces, a factor that is known to contribute to face recognition accuracy for same-race and cross-race targets [42, 43, 44, 45]. In addition, since the cross-race effect was not our research focus, we did not test European Canadian participants. As such, the present results regarding the cross-race effect may be confounded by stimulus properties (e.g., if East Asian faces were easier to recognize than the White faces because they were more distinctive), even though it remains unclear how stimulus effects, if any, could explain the discrepancies between the two studies. Through future research it would also be useful to determine more precisely the conditions under which a cross-race effect emerges among East Asian participants.

## Conclusions

In conclusion, the current research contributes to the literature on own-group bias in face recognition by demonstrating that whether people show improved face memory for strangers who share the same social group membership may hinge upon their cultural context and cultural heritage. People are likely to better recognize strangers who share a social group, but probably only after substantial exposure to and engagement in a Western cultural context.

## Supporting information

S1 Data Study 1 dataset. (CSV)

S2 Data Study 2 dataset. (CSV)

S3 Data Study 3 dataset. (CSV)

S1 File Study 1 code book. (CSV)

S2 File Study 2 code book. (CSV)

S3 File Study 3 code book. (CSV)

## References

[1] Hugenberg K, Wilson JP, See PE, Young SG. Towards a synthetic model of own group biases in face memory. Vis Cogn 2013;21(9–10):1392–1417. 10.1080/13506285.2013.821429

[2] Ng AH, Steele JR, Sasaki JY. Will you remember me? Cultural differences in own-group face recognition biases. J Exp Soc Psychol 2016;64:21–26. 10.1016/j.jesp.2016.01.003

[3] MacLin OH, Malpass RS. Racial Categorization of Faces: The Ambiguous Race Face Effect. Psychol Public Policy Law 2001;7(1):98–118. 10.1037/1076-8971.7.1.98

[4] Shriver ER, Young SG, Hugenberg K, Bernstein MJ, Lanter JR. Class, race, and the face: Social context modulates the cross-race effect in face recognition. Pers Soc Psychol Bull 2008;34(2):260–274. 10.1177/0146167207310455 18212334

[5] Bernstein MJ, Young SG, Hugenberg K. The cross-category effect: Mere social categorization is sufficient to elicit an own-group bias in face recognition. Psychol Sci 2007;18(8):706–712. 10.1111/j.1467-9280.2007.01964.x 17680942

[6] Tuettenberg SC, Wiese H. Intentionally remembering or forgetting own- and other-race faces: Evidence from directed forgetting. Br J Psychol in press. 10.1111/bjop.12413 31264716

[7] Baumeister RF, Leary MR. The need to belong: Desire for interpersonal attachments as a fundamental human motivation. Psychol Bull 1995;117(3):497–529. 10.1037/0033-2909.117.3.497 7777651

[8] Byrne D. The Attraction Paradigm. New York: Academic Press; 1971.

[9] Wilson JP, See PE, Bernstein MJ, Hugenberg K, Chartier C. Differences in anticipated interaction drive own group biases in face memory. PLoS ONE 2014;9(3). 10.1371/journal.pone.0090668 24599294 PMC3944439

[10] Snibbe AC, Kitayama S, Markus HR, Suzuki T. They saw a game: A Japanese and American (Football) Field Study. J Cross-Cult Psychol 2003;34(5):581–595. 10.1177/0022022103256480

[11] Falk CF, Heine SJ, Takemura K. Cultural variation in the minimal group effect. J Cross-Cult Psychol 2014;45(2):265–281. 10.1177/0022022113492892

[12] Heine SJ, Hamamura T. In search of East Asian self-enhancement. Pers Soc Psychol Rev 2007;11(2):204 10.1177/10888683070110020501 18453453

[13] Yuki M, Schug J. Relational mobility: A socio-ecological approach to personal relationships. In: Gillath O, Adams G, Kunkel AD. (eds.) Relationship Science: Integrating Evolutionary, Neuroscience, and Sociocultural Approaches. Washington, DC: Am Psychol Assoc; 2012 p.137–152.

[14] Li LMW, Adams G, Kurtiş T, Hamamura T. Beware of friends: The cultural psychology of relational mobility and cautious intimacy. Asian J Soc Psychol 2015;18(2):124–133. 10.1111/ajsp.12091

[15] Schug J, Yuki M, Horikawa H, Takemura K. Similarity attraction and actually selecting similar others: How cross-societal differences in relational mobility affect interpersonal similarity in Japan and the USA. Asian J Soc Psychol 2009;12(2):95–103. 10.1111/ajsp.2009.12.issue-210.1111/j.1467-839X.2009.01277.x

[16] Sznycer D, Takemura K, Delton AW, Sato K, Robertson T, Cosmides L, et al Cross-cultural differences and similarities in proneness to shame: An adaptationist and ecological approach. Evol Psychol 2012;10(2):352–370. 10.1177/147470491201000213 22947644 PMC3604996

[17] Thomson R, Yuki M, Talhelm T, Schug J, Kito M, Ayanian AH, et al Relational mobility predicts social behaviors in 39 countries and is tied to historical farming and threat. Proc Natl Acad Sci U S A 2018;115(29):7521–7526. 10.1073/pnas.1713191115 29959208 PMC6055178

[18] Yuki M, Sato K, Takemura K, Oishi S. Social ecology moderates the association between self-esteem and happiness. J Exp Soc Psychol 2013;49(4):741–746. 10.1016/j.jesp.2013.02.006

[19] Zhang R, Li LMW. The acculturation of relational mobility: An investigation of Asian Canadians. J Cross-Cult Psychol 2014;45(9):1390–1410. 10.1177/0022022114542850

[20] Sato K, Yuki M. The association between self-esteem and happiness differs in relationally mobile vs. stable interpersonal contexts. Front Psychol 2014;5(SEP). 10.3389/fpsyg.2014.01113 25346704 PMC4191559

[21] Yamagishi T, Yamagishi M. Trust and commitment in the United States and Japan. Motiv Emot 1994;18(2):129–166. 10.1007/BF02249397

[22] Takemura K. Being different leads to being connected: On the adaptive function of uniqueness in “open” societies. J Cross-Cult Psychol 2014;45(10):1579–1593. 10.1177/0022022114548684

[23] Falk CF, Heine SJ, Yuki M, Takemura K. Why do westerners self-enhance more than East Asians? Eur J Pers 2009;23(3):183–203. 10.1002/per.715

[24] Schug J, Yuki M, Maddux W. Relational mobility explains between- and within-culture differences in self-disclosure to close friends. Psychol Sci 2010;21(10):1471–1478. 10.1177/0956797610382786 20817913

[25] Sato K, Yuki M, Norasakkunkit V. A socio-ecological approach to cross-cultural differences in the sensitivity to social rejection: The partially mediating role of relational mobility. J Cross-Cult Psychol 2014;45(10):1549–1560. 10.1177/0022022114544320

[26] Lee S, Sobal J, Frongillo EA. Comparison of models of acculturation: The case of Korean Americans. J Cross-Cult Psychol 2003;34(3):282–296. 10.1177/0022022103034003003

[27] Ryder AG, Alden LE, Paulhus DL. Is acculturation unidimensional or bidimensional? A head-to-head comparison in the prediction of personality, self-identity, and adjustment. J Pers Soc Psychol 2000;79(1):49–65. 10.1037/0022-3514.79.1.49 10909877

[28] Goldberg LR. The Structure of Phenotypic Personality Traits. Am Psychol 1993;48(1):26–34. 10.1037/0003-066X.48.1.26 8427480

[29] Blair IV, Judd CM, Sadler MS, Jenkins C. The role of Afrocentric features in person perception: judging by features and categories. J Pers Soc Psychol 2002;83(1):5–25. 10.1037/0022-3514.83.1.5 12088132

[30] Gao W, Cao B, Shan S, Chen X, Zhou D, Zhang X, et al The CAS-PEAL large-scale Chinese face database and baseline evaluations. IEEE Trans Syst Man Cybern Pt A Syst Humans 2008;38(1):149–161. 10.1109/TSMCA.2007.909557

[31] Minear M, Park DC. A lifespan database of adult facial stimuli. Behav Res Methods Instrum Comput 2004;36(4):630–633. 10.3758/BF03206543 15641408

[32] Green DM, Swets JA. Signal Detection Theory and Psychophysics. Oxford, England: John Wiley; 1966.

[33] Hugenberg K, Young SG, Bernstein MJ, Sacco DF. The categorization-individuation model: An integrative account of the other-race recognition deficit. Psychol Rev 2010;117(4):1168–1187. 10.1037/a0020463 20822290

[34] Rhodes G. Configural Coding, Expertise, and the Right Hemisphere Advantage for Face Recognition. Brain Cogn 1993;22(1):19–41. 10.1006/brcg.1993.1022 8499110

[35] Hayes AF. Introduction to Mediation, Moderation, and Conditional Process Analysis: A Regression-Based Approach. New York, NY: The Guilford Press; 2013.

[36] Goh JX, Hall JA, Rosenthal R. Mini meta-analysis of your own studies: Some arguments on why and a primer on how. Soc Personal Psychol Compass 2016;10(10):535–549. 10.1111/spc3.12267

[37] Hedges LV, Vevea JL. Fixed- and random-effects models in meta-analysis. Psychol Methods 1998;3(4):486–504. 10.1037/1082-989X.3.4.486

[38] Mosteller FM, Bush RR. Selected quantitative techniques. In Lindzey G.(Ed.), Handbook of Social Psychology: Volume I. Theory and Method Cambridge, MA: Addison-Wesley; 1954.

[39] West AL, Zhang R, Yampolsky M, Sasaki JY. More than the sum of its parts: A transformative theory of biculturalism. J Cross-Cult Psychol 2017;48(7):963–990. 10.1177/0022022117709533

[40] Hayward WG, Rhodes G, Schwaninger A. An own-race advantage for components as well as configurations in face recognition. Cognition 2008;106(2):1017–1027. 10.1016/j.cognition.2007.04.002 17524388

[41] Michel C, Rossion B, Han J, Chung C, Caldara R. Holistic processing is finely tuned for faces of one's own race. Psychol Sci 2006;17(7):608–615. 10.1111/j.1467-9280.2006.01752.x 16866747

[42] Hancock KJ, Rhodes G. Contact, configural coding and the other-race effect in face recognition. Br J Psychol 2008;99(1):45–56. 10.1348/000712607X199981 17535471

[43] Meissner CA, Brigham JC. Thirty years of investigating the own-race bias in memory for faces: A meta-analytic review. Psychol Public Policy Law 2001;7(1):3–35. 10.1037/1076-8971.7.1.3

[44] Shepherd JW, Deregowski JB, Ellis HD. A Cross-Cultural Study of Recognition Memory for Faces. Int J Psychol 1974;9(3):205–212. 10.1080/00207597408247104

[45] Wright DB, Boyd CE, Tredoux CG. Inter-racial contact and the own-race bias for face recognition in South Africa and England. Appl Cogn Psychol 2003;17(3):365–373. 10.1002/acp.898

